# Pheochromocytoma as the first manifestation of MEN2A with RET mutation S891A: report of a case

**DOI:** 10.1007/s00595-013-0826-8

**Published:** 2014-01-22

**Authors:** Yatsuka Hibi, Tamae Ohye, Kimio Ogawa, Yoshimi Shimizu, Masahiro Shibata, Chikara Kagawa, Yutaka Mizuno, Shinya Uchino, Shinji Kosugi, Hiroki Kurahashi, Katsumi Iwase

**Affiliations:** 1 Department of Endocrine Surgery, Fujita Health University School of Medicine, 1-98 Dengakugakubo, Kutsukake-cho, Toyoake, Aichi 470-1192 Japan; 2 Division of Molecular Genetics, Institute for Comprehensive Medical Science, Fujita Health University, Toyoake, Aichi 470-1192 Japan; 3 Department of Surgery, Noguchi Thyroid Clinic and Hospital Foundation, Beppu, Oita 874-0932 Japan; 4 Department of Medical Ethics and Medical Genetics, Kyoto University Graduate School of Public Health, Kyoto, 606-8501 Japan; 5 MEN Consortium of Japan, Kyoto, Japan

**Keywords:** MEN2A, RET, Pheochromocytoma

## Abstract

We report a rare case with pheochromocytoma as the first manifestation of multiple endocrine neoplasia type 2A with *RET* mutation S891A. Bilateral pheochromocytomas were identified in a 54-year-old woman. Screening for RET revealed a rare S891A mutation located in the intracellular tyrosine kinase domain. This mutation was previously recognized as one of the mutations only in cases manifesting solely medullary thyroid carcinomas (MTCs). Since calcitonin stimulation test indicated positive result, total thyroidectomy was performed 1 year after the bilateral adrenalectomy, and C-cell hyperplasia was diagnosed by histopathological examination. Our report suggests that cases with S891A mutation, akin to those with other *RET* mutations, require screening for pheochromocytoma. In addition, it is indicated that calcitonin stimulation test should be performed even in the unaffected elder cases with S891A mutation although the mutation is classified as lowest risk group on MTC in guidelines.

## Introduction

Multiple endocrine neoplasia type 2 (MEN2) is an autosomal, dominantly inherited disorder manifesting various combinations of medullary thyroid carcinoma (MTC) and pheochromocytoma, with hyperparathyroidism (MEN2A) or neuromas of the enteric autonomic nerve cells (MEN2B). MEN2 is caused by the gain-of-function mutation in the *RET* protooncogene, encoding a transmembrane receptor tyrosine kinase [1, 2]. Most of the mutations, found in the cysteine-rich extracellular domain, give rise to ligand-independent receptor dimerization and cross-phosphorylation, leading to constitutive activation of the downstream signal of the receptor [3]. Mutation of the cysteine codon 634 constitutes 80–90 % of MEN2A cases, although those caused by mutations of the cysteine codon 611, 618, and 620 are also observed. Although they are a minor subset, *RET* mutations in MEN2A cases are also identified within the intracellular domain, including those originally reported as mutations of familial medullary thyroid carcinoma (FMTC). On the other hand, most cases with MEN2B carry M918T or A883F mutations in the tyrosine kinase domain, suggesting strong genotype–phenotype correlations.

The discovery of strong genotype–phenotype correlations that govern the development of MEN2A-associated endocrine neoplasia in MEN2 cases has prompted us to utilize the identified *RET* mutations for the prediction of prognosis, and for the determination of surgical concept. In particular, mutations at codons 609, 768, 790, 791, 804 and 891 are classified as level 1, having the lowest risk for aggressiveness among the three levels of MTC [4]. A rare mutation, S891A, has been associated solely with intermediate-risk FMTC [5–7]. For the carriers of such FMTC mutations, intensive screening for age-related development of pheochromocytoma need not be started until they are 20 years old [7]. However, a rare case with a S891A mutation expressing MTC and pheochromocytoma was recently reported, suggesting the limitation of genotype-based predictions [8].

In this manuscript, we report a rare case of a patient who was affected by bilateral pheochromocytomas as the first manifestation of MEN2A, whose subsequent screening for *RET* mutation identified S891A.

## Case report

**Fig. 1:**
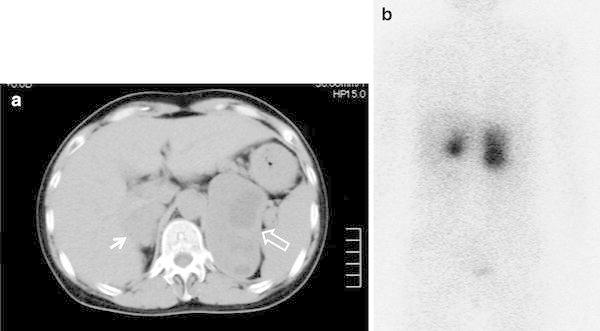
**a** Computed tomography of abdomen shows *right* (*arrow*) and *left* (*open arrow*) adrenal tumors. **b** ^131^I-MIBG scan reveals abnormal uptakes indicating bilateral pheochromocytomas

A 57-year-old woman visited a local hospital with cough and vomiting. She had episodes of periodic headaches and paroxysmal palpitations. Bilateral adrenal tumors were identified by abdominal CT scan (maximum diameter of 4 cm for right mass and 9 cm for left mass) (Fig. 1a). Pheochromocytomas were diagnosed by ^131^I-metaiodobenzylguanidine (MIBG) scan (Fig. 1b), along with elevated urinary catecholamine and metabolite concentrations (Table 1), and she was, therefore, transferred to the Fujita Health University Hospital for surgical management. On admission, blood pressure was 111/68 mmHg. Since her maternal aunt had adrenal disease and had died from cerebral vascular disease, MEN2A was strongly suspected. Although her basal serum calcitonin level was normal(37.0 pg/ml), elevated levels were recorded following stimulation with 2 mg/kg of Ca^2+^ infusion (330 pg/ml). However, neither ultrasonography nor ^99m^Tc-methoxy-isobutyl-isonitrile (MIBI) scan showed any cervical lesions. Although serum PTH level was elevated (340.5 pg/ml), serum calcium and phosphorus were within normal range (9.3, 3.8 mg/dl), and urinary Ca excretion was not elevated (Ca/creatinine = 0.163). Finally, screening for the *RET* gene was performed after appropriate informed consent was obtained (approved by the Ethical Review Board for Human Genome Studies at Fujita Health University), and, unexpectedly, the S891A mutation was identified.

**Table 1:** Urinary catecholamine and metabolite level

	mg/day	(Normal range)
Adrenaline	961.6	(3.4–26.9)
Noradrenaline	180.6	(48.6–168.4)
Dopamine	1247.8	(365.0–961.5)
VMA	43.1	(1.5–4.3)
HVA	7.1	(2.1–6.3)
Metanephrine	23.02	(0.04–0.19)
Normetanephrine	4.96	(0.09–0.33)

**Fig. 2:**
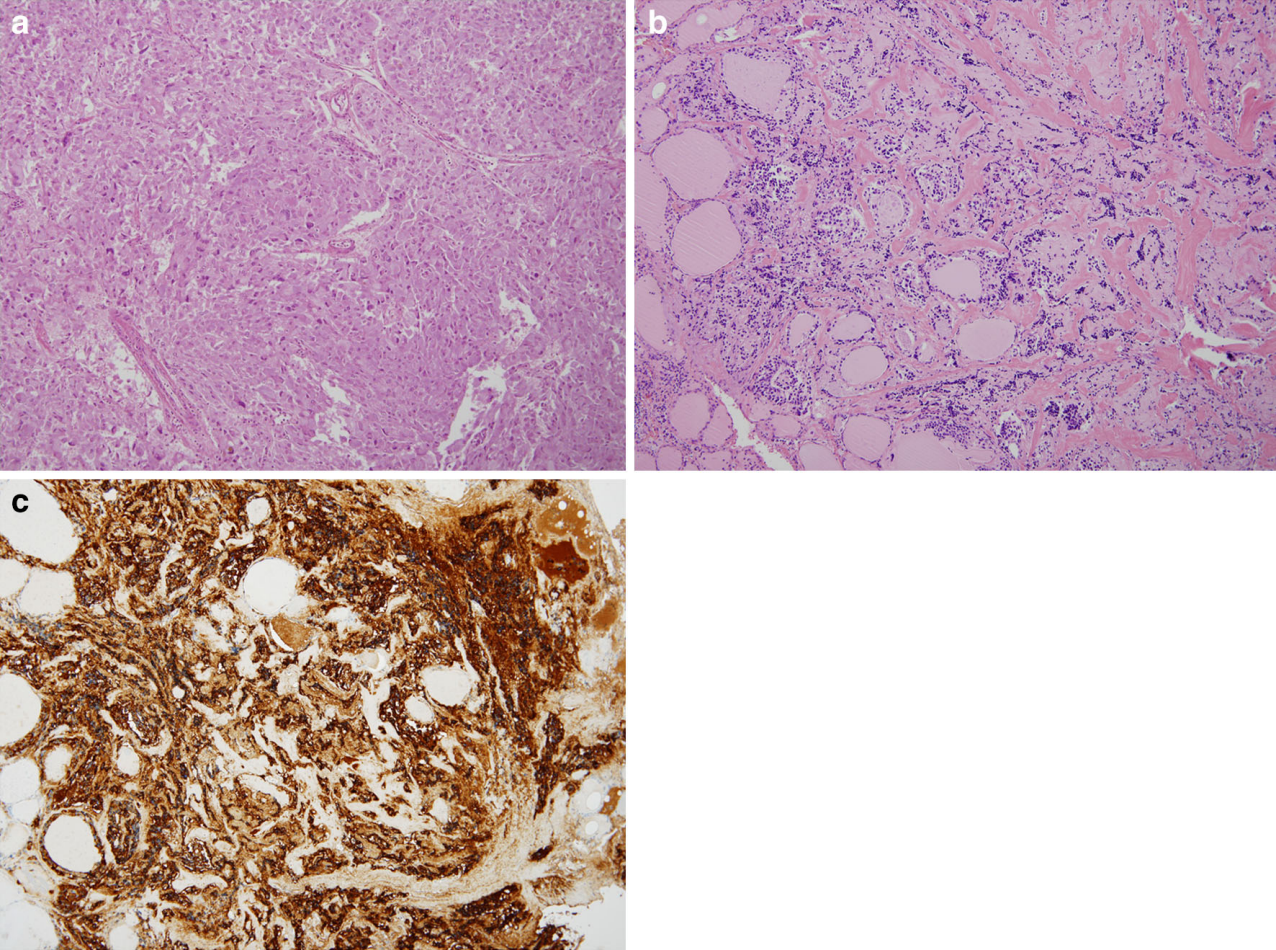
**a** Microscopy of *left* adrenal tumor shows pheochromocytoma (hematoxylin & eosin, ×100). **B** Microscopy of thyroid gland shows C-cell hyperplasia (hematoxylin &eosin, ×100). **c** Microscopy of thyroid gland shows C-cell hyperplasia (calcitonin immunostaining, ×100)

**Fig. 3:**
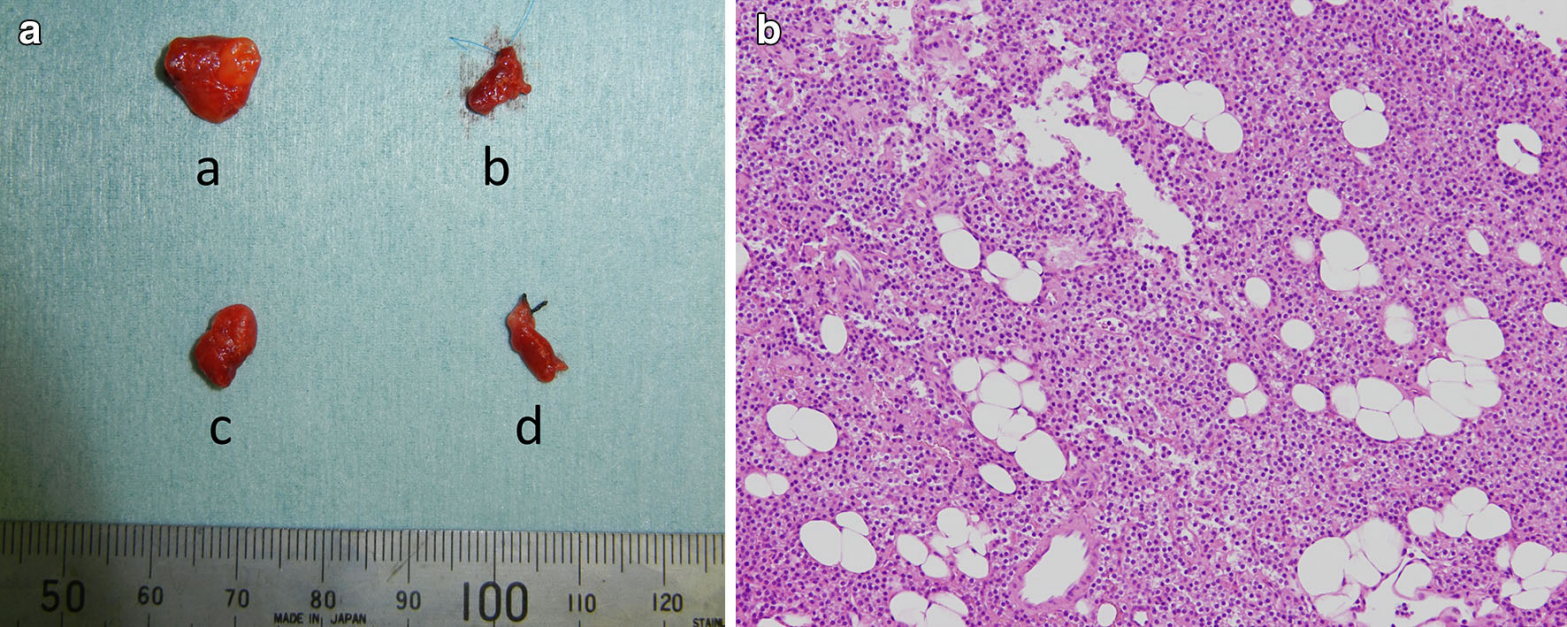
**a** Macroscopy of the resected parathyroid glands shows slightly enlarged *right upper* and *lower* gland. (**a** *right upper* gland, **b** *left upper* gland, **c** *right lower* gland, **d** *left lower* gland) **b** Microscopy of right upper parathyroid gland shows no hyperplasic change (hematoxylin & eosin, ×100)

Bilateral adrenal tumors were surgically resected, and the diagnosis of pheochromocytomas was confirmed by histological examination (Fig. 2a). One year later, the patient underwent prophylactic total thyroidectomy. Histological examination demonstrated that multiple nodal lesions were scattered, indicating the presence of C-cell hyperplasia without any evidence of MTC (Fig. 2b, c). Regarding the four resected parathyroid glands (Fig. 3a), two right glands were slightly enlarged (231 mg for superior and 118 mg for inferior glands) compared with the two left glands (48 mg for superior and 15 mg for inferior glands). However, no specific histological change was observed even in the enlarged glands (Fig. 3b). Approximately, one-third of the resected parathyroid glands were implanted into the muscle of the left forearm.

**Fig. 4:**
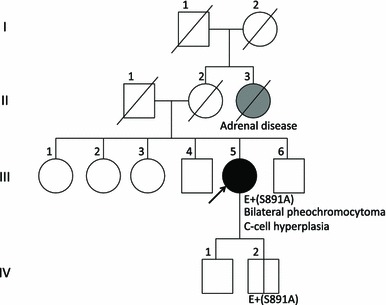
Pedigree of the family. The *arrow* indicates the proband. The II-3, who was affected by adrenal disease and died from cerebral vascular disease, might possibly carry the S891A mutation

Currently, the patient’s two brothers (59 and 53 years old, respectively) and three sisters (69, 64 and 61 years old, respectively) do not have any clinical symptoms associated with MEN2A, although screening for *RET* mutation has not been performed for them yet. The patient has two sons, 29 and 27 years old, neither of whom have clinical symptoms. The 27-year-old son requested *RET* mutation screening, and indeed, the S891A mutation was identified (Fig. 4). Routine chemical screening of the blood, including basal serum calcitonin levels, was all normal. Ultrasonography did not detect any mass within the thyroid. He is being followed up carefully as a presymptomatic MEN2A case.

## Discussion

S891A mutation constitutes 2 % of all *RET* mutations identified in MEN2/FMTC cases [9]. Early reports stress the association of this mutation with FMTC, but accumulating evidence shows the mutant’s capacity to induce a wider spectrum of MEN2A [10]. S891A mutation causes MTC in 63.5 % of cases, pheochromocytoma in 4.1 % of cases and parathyroid hyperplasia in 4.1 % of cases [10]. Compilation of MEN2A-related clinical manifestation in patients with RETS891A mutation in previous reports [5, 6, 8, 10–17] is described in Table 2. Indeed, the management guideline of medullary thyroid cancer by the American Thyroid Association categorizes pheochromocytoma in S891A mutation as ‘rare’ [18]. None of ten Japanese cases with S891A mutation reported in a recent study had pheochromocytoma [17], but our case report combined with previous data indicates that S891A patients as well as other MEN2A patients require early detection of subclinical pheochromocytoma to prevent a potential hypertensive crisis In MEN2 patients, the gain-of-function mutation in the *RET* receptor tyrosine kinase gene constitutively activates the downstream signals, leading to transformation of the cells [3]. Although the mutations in the cysteine-rich extracellular domain all target cysteine codons, inducing ligand-independent RET dimerization, the mutations located in the intracellular tyrosine kinase domain do not target cysteine codons. These mutations, including S891A, are considered to give rise to structural changes in the protein facilitating the access of adenosine triphosphate and substrate to the catalytic site [19]. The less constitutive RET kinase activation relative to mutations in the cysteine-rich extracellular domain might result in less neoplastic transforming capacity [20, 21].

**Table 2:** MEN2A-related clinical manifestation in patients with RETS891A mutation in previous reports

	Total patients	No. affected patients	No. asymptomatic gene carriers	Mean age at Dx (year)	MTC	CCH	PH	PHEO
Schulte et al. [10]	36	33	3	41	25	23	3	1
Jimenez et al. [8]	6	3	3	45	2	2	0	1
Hofstra et al. [5]	5	3	2	47	3	1	0	0
Dang et al. [6]	3	3	0	ND	3	ND	ND	ND
Elisei et al. [11]	14	6	8	44	6	ND	ND	ND
Yip et al. [12]	3	3	0	ND	3	DC	0	0
Asari et al. [13]	1	1	0	ND	1	0	0	1
Paszko et al. [14]	2	2	0	ND	2	0	0	0
Wohllk et al. [15]	4	2	2	49	2	2	0	0
Frank-Raue et al. [16]	5	5	0	ND	1	4	ND	ND
Imai et al. [17]	10	ND	ND	ND	ND	ND	ND	0
Total	89	61	18		48	32	3	3

Modified Table 2 in [10]

*DC* data clustered, *Dx* diagnosis, *ND* no data, *MTC* medullary thyroid carcinoma, *CCH* C-cell hyperplasia, *PH* primary hyperplasia, *PHEO* pheochromocytoma

The S891A mutation is classified as level 1 [4] or level A [18], the lowest risk group among the three (level 1–3) or the four (level A–D) *RET* codon mutation stratification categories. The penetrance and aggressiveness of MTC arising in cases with S891A are variable, but MTC develops at a later age and grows more slowly than with the higher risk mutations. There has been little consensus concerning the management of patients with level 1/level A mutations. According to the guidelines, cases with S891A mutation still need prophylactic resection, and some experts recommended thyroidectomy by the age of 5 years, while others suggest that thyroidectomy by the age of 10 years is appropriate with careful follow-up and periodic calcitonin testing [4, 14, 18]. Since the timing of thyroidectomy should be determined considering the earliest finding of MTC in asymptomatic carriers, prophylactic thyroidectomy at an early age is generally recommended even in cases with level 1/level A mutations [14, 16, 18, 22]. In this regard, two S891A cases, whose MTCs were diagnosed at 17 years old, might be of help in determining the timing of surgery [10].

In this patient, serum levels of PTH were mildly elevated, although the urinary excretion of Ca^2+^ was not increased. One possibility for this is that the patient has primary hyperparathyroidism due to parathyroid gland hyperplasia or adenoma, as is observed in 20–30 % of MEN2A cases [23]. Since the resected right parathyroid glands of the patient were found to enlarge, we carefully examined the histology of the four resected parathyroid tissues, but there was no evidence of hyperplasia or adenoma. Alternatively, it is possible that the elevated serum PTH levels were due to secondary to decreased serum Ca^2+^ levels induced by excess calcitonin excretion from the C-cell hyperplasia. Furthermore, this hypothesis implies that the patient with the S891A mutation carried slow-growing C-cell hyperplasia for a long period, during which time the decreased Ca^2+^ was compensated for by the secondary hyperparathyroidism. These data lend support to the concept that C-cell hyperplasia in the patient with S891A mutation, in spite of its slow growth speed, has a high chance of obtaining another hit for malignant transformation. The patient’s two brothers and three sisters, all of whom are more than 50 years old, do not have any clinical symptoms associated with MEN2A at this moment. This implies that early surgical intervention is not required for S891A cases, although it is still possible that none of the brothers and sisters carries the S891A mutation. Careful follow-up of these older relatives is required even though they do not yet have any MEN2-related symptom.
